# Phylogeny and expression patterns of ERF genes that are potential reproductive inducers in hybrid larch

**DOI:** 10.1186/s12864-024-10188-3

**Published:** 2024-03-18

**Authors:** Junfei Hao, Daixi Xu, Chen Wang, Qing Cao, Qingrong Zhao, Miaomiao Xie, Hanguo Zhang, Lei Zhang

**Affiliations:** State Key Laboratory of Tree Genetics and Breeding, Northeast Forestry University, 150040 Harbin, China

**Keywords:** Larch, ERF family, Phylogenetic analysis, Reproductive development

## Abstract

**Background:**

Larch is an important component of northern forests and a major cultivated tree species in restoration of forest cover using improved seed material. In recent years, the continuous low seed production has severely affected the production of improved variety seedlings and natural regeneration. However, research on the reproductive growth of gymnosperms is extremely scarce.

**Results:**

In this study, based on differential transcriptome analysis of two asexual reproductive phases, namely high-yield and low-yield, we further screened 5 ERF family genes that may affect the reproductive development of larch. We analyzed their genetic relationships and predicted their physicochemical properties. The expression patterns of these genes were analyzed in different tissues, developmental stages, hormone treatments, and environmental conditions in hybrid larch.

**Conclusion:**

The results showed that all 5 genes were induced by low temperature and ABA, and their expression patterns in different tissues suggested a suppressive role in the development of female cones in larch. Among them, *LkoERF3-like1* and *LkoERF071* may be involved in the flowering age pathway. This study enriches the scarce research on reproductive development in gymnosperms and provides a theoretical basis and research direction for regulating the reproductive development of larch in seed orchards.

## Background

Larch (*Larix* spp.) is one of a dominant species in the boreal coniferous forest and serves as a primary afforestation species in northern China due to its favorable growth and wood properties. The establishment of larch stand using improved seed material is an essential initiative for restoring forest ecology in the region. Currently, afforestation efforts rely heavily on high-quality seeds produced in seed orchards. However, the limited and inconsistent seed yield from these orchards is a significant constraint for planned afforestation. To address this issue, a comprehensive study on the reproduction mechanism of larch is necessary. In higher plants, the flowering induction process is coordinated through multiple pathways, influenced by both endogenous factors and environmental cues. Among these pathways, light [1, 2] and temperature [3, 4] are the primary external signals. While angiosperms have well-defined flowering pathways such as the photoperiod pathway [5, 6], temperature pathway [7, 8], gibberellin pathway [9], autonomous pathway [10], and age pathway [11], similar pathways exist in gymnosperms with potential variations [12–15].

**Fig. 1:**
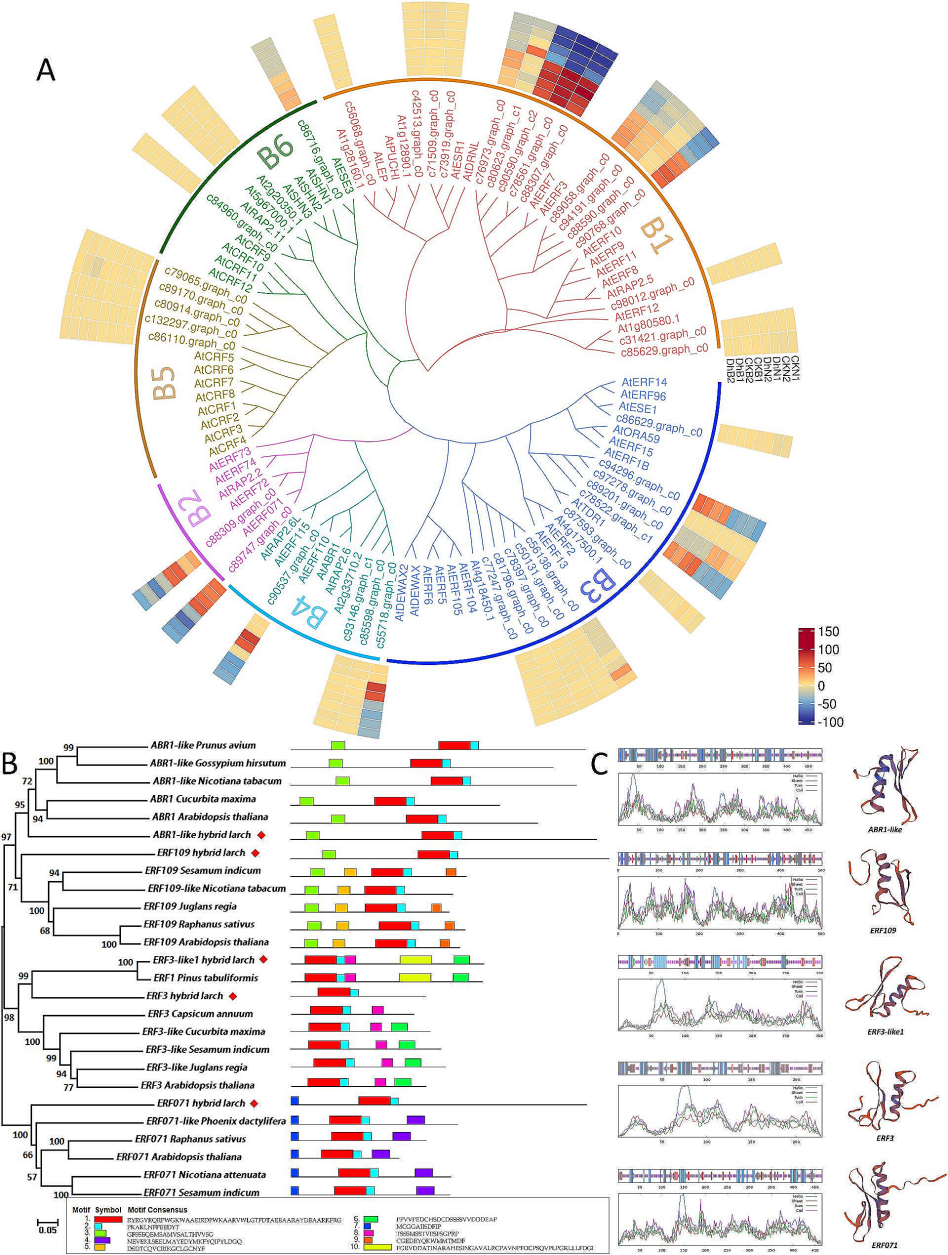
Phylogenetic analysis **(A)** of ERF family genes during the formation period of larch strobilus buds, with data from previous studies(CKN: Needles of low yield clones, DhN: Needs of high yield clones, CKB: Buds of low yield clones, DhB: Buds of high yield clones); motif identification **(B)**, and prediction of secondary and tertiary structures **(C)** of five ERF genes

The Ethylene Response Factor (ERF) belongs to the AP2/ERF family and contains a conserved ERF domain of approximately 58–59 amino acids. Previous studies have demonstrated that genes in this family respond to both biotic [16] and abiotic [17, 18] stresses by integrating signals from multiple pathways, including jasmonic acid, ethylene, and salicylic acid pathways, thereby finely regulating defense responses [19]. The ERF family’s ability to respond to environmental signals has led to an increasing number of reports in recent years (Table 1) concerning its involvement in plant flowering regulation by integrating external signals [20–23]. The study of the reproduction mechanism in coniferous trees, characterized by their elongated juvenile periods and extensive genomes, remains insufficiently understood. The hybrid larch in this study is an early artificial hybrid cultivar in China, combining the superior traits of both L. kaempferi and L. olgensis. This hybrid exhibits remarkable growth rates and premium wood quality due to the combination of the genomes from both parent species. As a result of this genetic integration, its reproductive growth patterns and phenological rhythms display characteristics inherited from both parents, rendering it an excellent subject for research into the reproductive development of larch species in northern areas. In earlier investigations, we identified the flowering induction period of hybrid larch through anatomical sections and observed a significant enrichment of AP2 family genes during this period [24]. Notably, five ERF-like genes exhibited significant differences in expression among distinct reproductive materials(Figure 1A), suggesting their potential role in the flowering induction of larch. Following CDS (coding region) screening and identification of conserved domains, we obtained five full-length genes. In this study, we conducted bioinformatics and expression pattern analyses of these five genes to elucidate their evolutionary and reproductive roles, aiming to enhance our understanding of reproduction regulation in gymnosperms and improve the fruiting performance of seed orchards.

**Table 1:** ERF genes are involved in flowering in some species

Species	Gene name	Function of flowering	Reference
*Chrysanthemum morifolium*	*CmERF110*	Promoting	[25]
*Chrysanthemum morifolium*	*CmERF3*	Delay	[26]
*Solanum lycopersicum*	*SIERF36*	Promoting	[27]
*Arabidopsis thaliana*	*AtERF1*	Delay	[28]
*Glycine max*	*TOE4b*	Promoting	[20]
*Lilium longiflorum*	*LlERF110*	Delay	[29]

## Results

### Identification and sequence feature analysis of 5 ERF genes

Flower induction is the main factor affecting the yield of larch seeds. In previous studies, we identified 5 ERF genes as potential flower inducing genes in larch [24]. To further identify these 5 ERF genes, we searched the UniProt database using HMM and BLAST algorithms and found 21 similar sequences from other plants (Fig. 1B). The phylogenetic tree constructed using the Neighbor-Joining method clearly divided these 26 genes into 4 major branches. Based on this classification, the 5 ERF genes in hybrid larch were named *ABR1-like* (*c90537.graph_c0*, GenBank: PP317119), *ERF109* (*c55718.graph_c0*, GenBank: PP317120), *ERF3-like1* (*c88590.graph_c0*, GenBank: PP317122), *ERF3* (*c76973.graph_c0*, GenBank: PP317123), and *ERF071* (*c88309.graph_c0*, GenBank: PP317121). Within each main branch, the larch genes were distantly related to genes from other plants, indicating significant divergence between the ERF transcription factor families of gymnosperms and angiosperms. It is worth noting that within the *ERF3* branch, *ERF3-like1* showed high similarity to *ERF1* in *Pinus tabuliformis*, suggesting that gene differentiation within gymnosperms is not pronounced. Motif analysis revealed that these 26 genes all possessed the typical AP2 domain and could be further categorized into 4 classes, with high similarity of motifs within each category.

Through online software analysis, the physicochemical properties of these 5 ERF genes were investigated. The results (Table 1) showed that *ERF3* and *ERF3-like1* have fewer amino acids, consisting of 229 and 304 AAs respectively, while the other 3 genes have amino acid counts ranging from 466 to 500 AAs. The molecular weight ranges from 2.48 kDa (*ERF3*) to 5.44 kDa (*ERF109*), corresponding to the size of the proteins in relation to the amino acid count. The theoretical isoelectric point of *ERF109* and *ERF071* is less than 7, indicating acidic proteins, while *ABR1-like* has an isoelectric point close to 7, suggesting a neutral protein. The remaining 2 genes are classified as basic proteins. Furthermore, secondary and tertiary structure prediction (Table 2; Fig. 1C) suggested that all these genes encode hydrophilic proteins. The ranges of alpha helix, extended strand, and random coil were found to be 19.53%$\sim$37.06%, 7.45%$\sim$18.03%, and 51.32%$\sim$62.45%, respectively.

**Table 2:** Analysis of the physicochemical properties of five ERF family proteins

Gene ID	Physicochemical property analysis					The secondary structure analysis			
	Number of amino acids	Molecular weight	Theoretical pI	Instability index		Grand average of hydropathicity	Alpha helix	Extended strand	Random coil
*ABR1-like*	483	54106.98	7.13	77.57		-0.940	37.06%	7.45%	55.49%
*ERF109*	502	54427.95	6.10	55.11		-0.616	27.49%	14.54%	57.97%
*ERF3-like1*	304	32961.73	8.78	48.78		-0.556	30.92%	17.76%	51.32%
*ERF3*	229	24826.91	9.30	52.85		-0.440	29.69%	12.66%	57.64%
*ERF071*	466	50978.42	5.04	41.23		-0.569	19.53%	18.03%	62.45%

### Gene expression patterns at different developmental stages and tissues

**Fig. 2:**
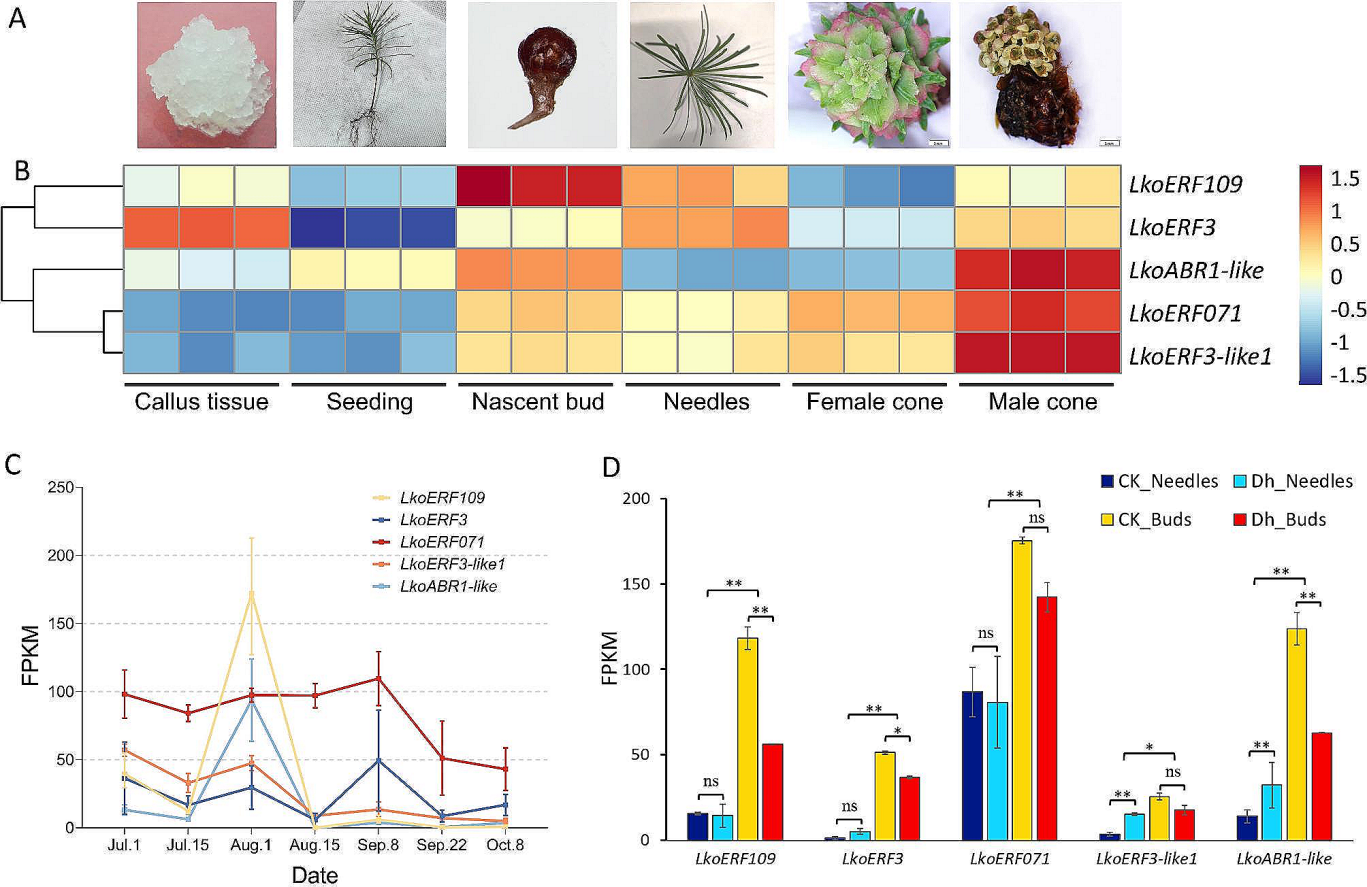
Pictures of different tissue parts of larch **(A)**; Expression levels of 5 ERF genes in different tissues **(B)**, developmental stages **(C)**, and clones **(D)**. The relative expression data was obtained by calculating qRT-PCR data using the 2^−ΔΔCT^ method, and relative expression heatmaps were plotted for different tissue parts after Z-source standardization

Reproductive growth is the most crucial stage in perennial plant’s life cycle, involving both the transition from seedling to adulthood and the phase changes from flowering determination to flower bud formation in adult plants. To examine if these genes have an adult-specific effect, expression pattern analysis was carried out in various tissue types (Fig. 2A), including embryogenic callus, 90-day-old seedlings, undifferentiated shoot apices of adult plants, needle buds, and female and male cones (Fig. 2B). The results revealed that the expression levels of *ERF3-like1* and *ERF071* were significantly higher in different parts of adult plants compared to callus and seedlings. *ERF109* showed the highest expression level in undifferentiated shoot apices, while *ERF3* exhibited the highest expression level in callus tissue. Except for *ERF3*, the expression levels of the other four genes showed minimal variation between callus tissue and seedlings. It is worth noting that all genes exhibited higher expression in male cones compared to female cones. Among them, *ABR1-like*, *ERF3-like1*, and *ERF071* exhibited the highest expression level in male cones.

Flower induction marks the beginning of the reproductive development cycle in adult plants. In previous studies [24], we found that the flower induction period of local larch trees in this region occurs from late July to early August, followed by approximately two months of strobili organ development. To investigate the performance of ERF genes during reproductive bud development, reproductive buds were collected at seven different time points, starting from when the new shoots emerged in early July until just before winter, and gene expression analysis was conducted (Fig. 2C). The results showed that the expression patterns of *ABR1-like* and *ERF109* were consistent across the seven time points, with the highest expression levels observed in early August and very low expression levels observed after August 15th. *ERF3-like1* exhibited higher expression levels before early August, followed by lower expression levels from August to early October. *ERF071* showed higher expression levels before early September, followed by lower expression levels. The expression level of *ERF3* showed continuous fluctuations throughout the experiment. These 5 genes showed high consistency in expression levels between different clones of larch during the flowering induction period: the expression levels in buds were higher than those in needles (Fig. 2D), and the expression levels in buds of multi seed setting clones (Dh_Buds) were lower than those of low seed setting clones (CK_Buds).

### Changes in expression levels of 5 ERF genes after phytohormone treatments

**Fig. 3:**
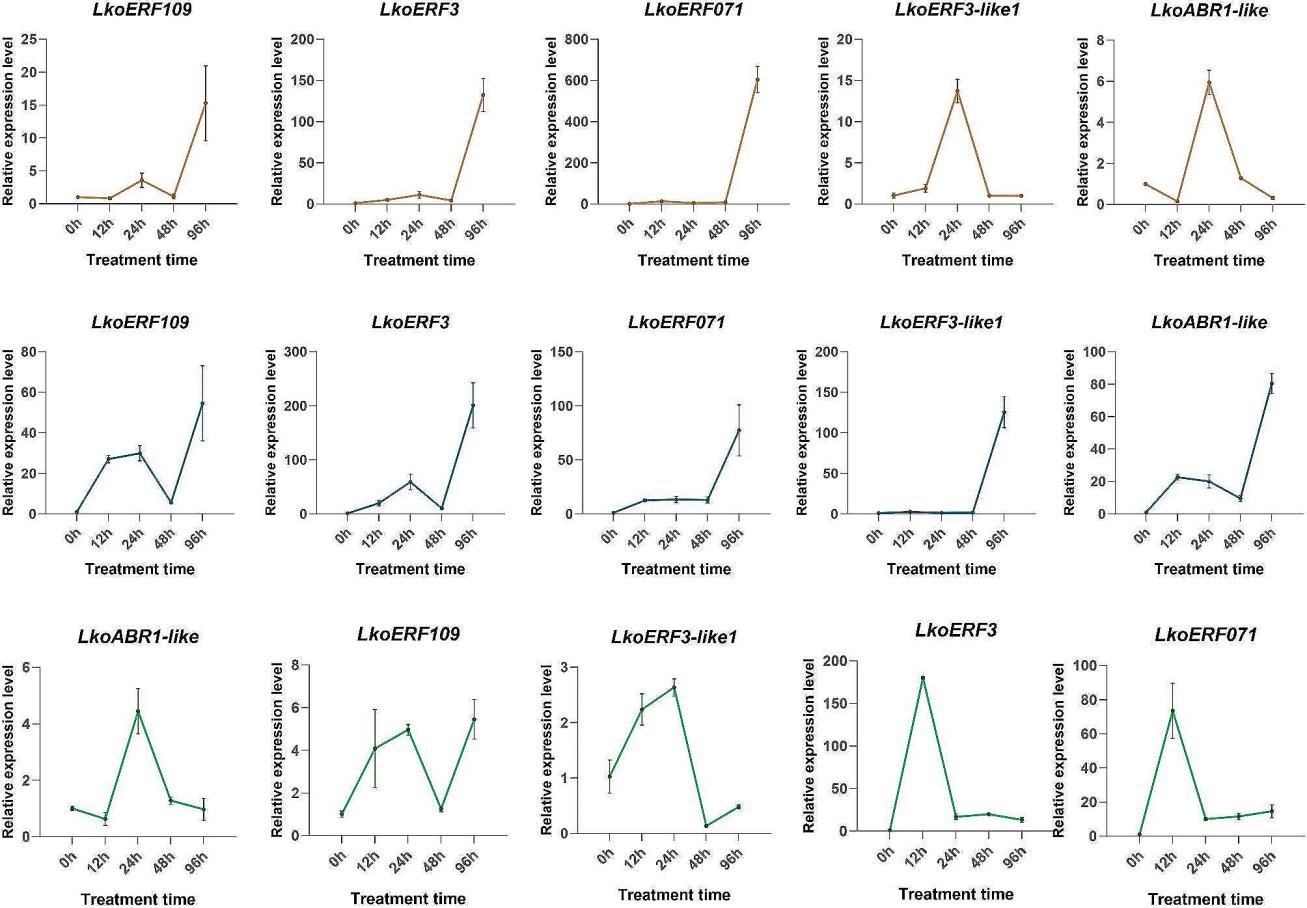
The relative expression levels of genes at different times after hormone treatment. The yellow, blue, and green lines represent the gene expression levels under GA_3_, ABA, and IAA treatments, respectively. The relative expression level was calculated using the 2^−ΔΔCT^ method

A large body of research has demonstrated the significant role of phytohormones in the signal integration and regulation of flower bud development in higher plants [30]. To investigate the response of these five ERF genes to hormones, we conducted a study on the gene expression changes under hormone treatments. The results (Fig. 3) revealed that the five genes can be divided into two categories after gibberellin (GA) treatment. *ABR1-like* and *ERF3-like1* showed similar expression patterns: an increase in expression levels 24 h after GA treatment followed by a decrease to the pre-treatment level. In contrast, *ERF109*, *ERF071*, and *ERF3* showed a slight increase in expression after 24 h of treatment, followed by a significant increase at 96 h. Regarding abscisic acid (ABA) treatment, all the genes exhibited a similar trend. *ABR1-like*, *ERF109*, *ERF3*, and *ERF071* showed an initial increase in expression levels within 12 to 24 h, followed by a decrease, and then reaching the highest expression level at 96 h. However, LkoERF3-like1 showed little variation before 48 h of treatment, and a significant increase in expression levels at 96 h. The expression patterns of the five genes under indole-3-acetic acid (IAA) treatment can be classified into two categories. *ABR1-like*, *ERF109*, and *ERF3-like1* reached their highest expression levels 24 h after treatment, followed by a decrease, albeit with minimal changes. On the other hand, *ERF3* and *ERF071* exhibited consistent patterns: a sharp increase in expression within 12 h of treatment, followed by a sustained high level (approximately 10 times higher than the initial level).

### Response to photoperiod and temperature

**Fig. 4:**
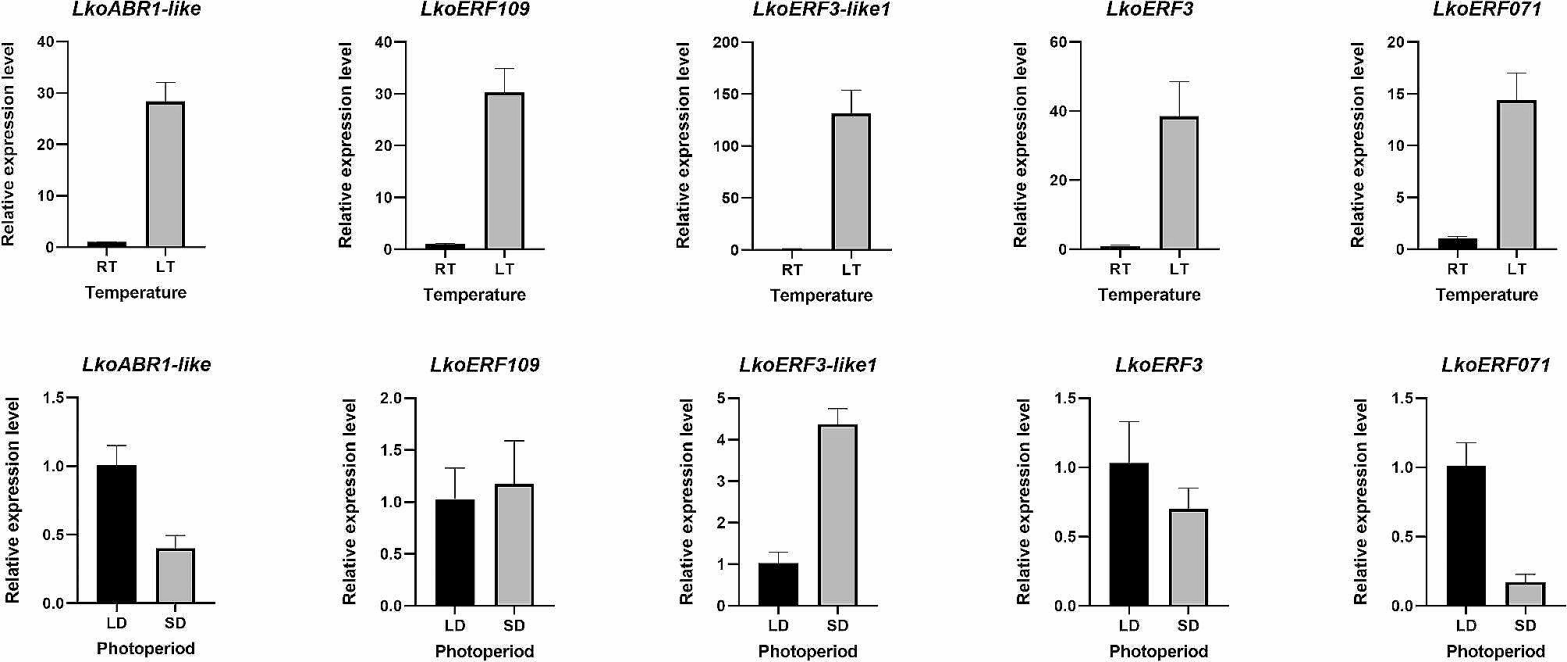
The relative expression levels of 5 ERF genes under different photoperiod and temperature conditions. The relative expression level was calculated using the 2^−ΔΔCT^ method

Both light and temperature play crucial roles in the photoperiodic pathway, vernalization, and temperature pathway governing flowering in higher plants. To investigate the response of these five genes to photoperiod and temperature, we performed quantitative analysis of their expression levels in seedlings subjected to different treatments. The results (Fig. 4) demonstrate the following findings: *ABR1-like* and *ERF071* exhibit an upregulation in expression in response to long-day photoperiods, while *ERF3-like1* shows a response to short-day photoperiods. On the other hand, *ERF109* and *ERF3* are not sensitive to changes in photoperiod. Regarding temperature response, all five genes exhibit a consistent behavior, showing an upregulation of expression induced by low temperature.

### Prediction of the interaction network of five ERF genes involved in flowering induction in larch

**Fig. 5:**
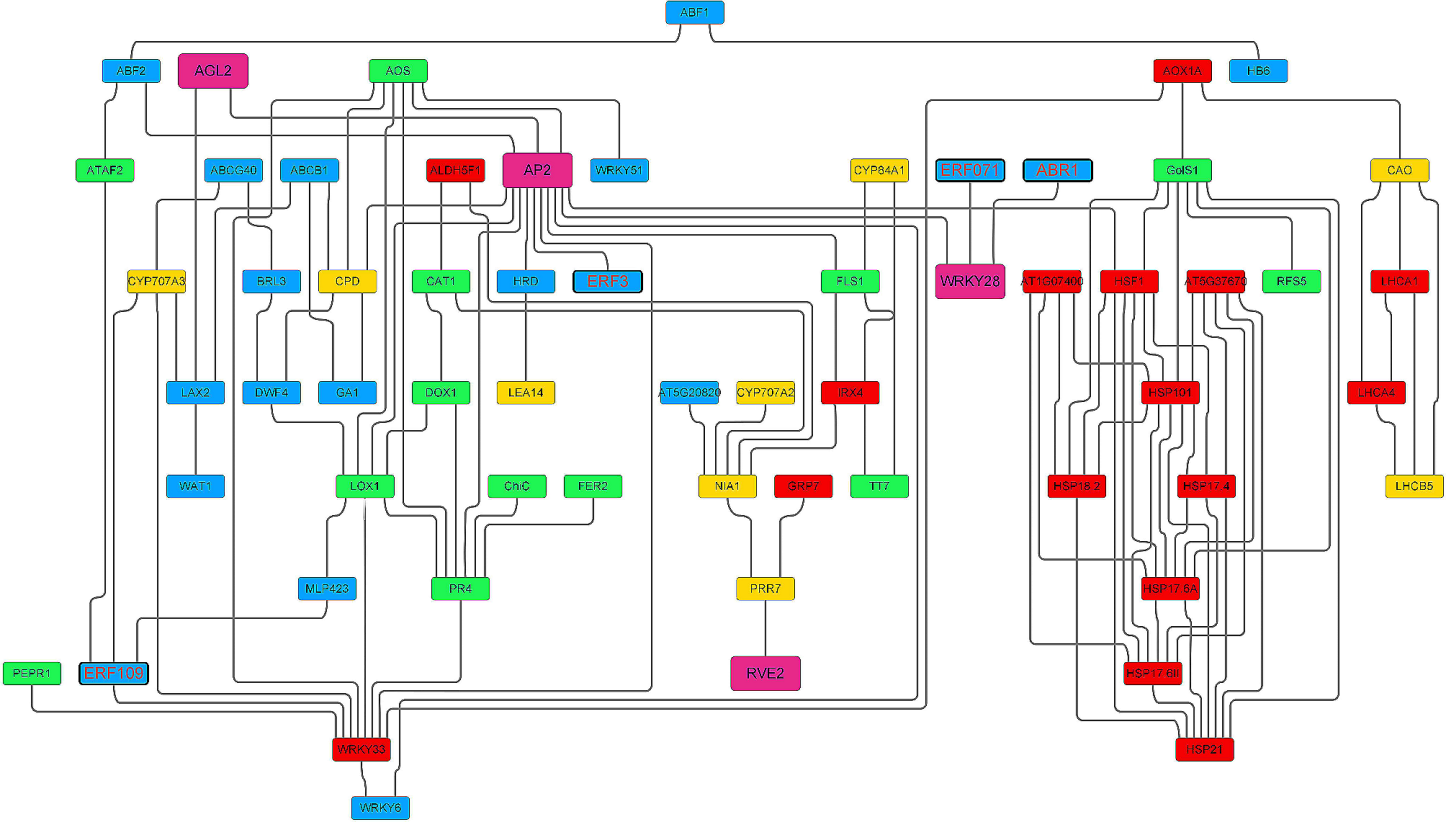
Shows the predicted interacting network of 5 ERF genes involved in reproductive development in hybrid larch. Green represents the response to hormones (GO:0009725), blue represents hormone-mediated signaling pathways (GO:0009755), red represents temperature response (GO:0009266), yellow represents response to light stimulation (GO:0009416), and magenta represents genes related to reproductive development

In our previous study, we identified 2893 differentially expressed genes, including these 5 ERF genes, in the shoot tips of two larch clones with contrasting flowering ability, namely high fertility and low fertility. The most significantly enriched Gene Ontology (GO) term was “response to stimulus” [24]. To analyze the roles of these 5 genes in the induction of flowering, we conducted an interaction network prediction using the STRING database. The results (Fig. 5) revealed that these 5 genes were annotated to the GO term “hormone signal transduction” and showed proximity to genes related to reproductive development. Specifically, *LkoABR1-like* and *LkoERF071* were predicted to interact with the flowering gene *AP2* through *WRKY28*, while *LkoERF3* and *LkoERF3-like1* may directly interact with floral homeotic gene *AP2*.

## Discussion

Reproduction is an eternal topic in biology, and the differences in reproductive mechanisms form the basis of species diversification. While significant differences exist in the reproductive organs between gymnosperms and angiosperms, the mechanisms underlying flower induction and flowering rhythms are relatively similar [31, 32]. In this study, the five ERF genes can be roughly divided into four branches, with relatively high sequence conservation within each branch among different species. This suggests that the differentiation of ERF family genes may have occurred long before the divergence between gymnosperms and angiosperms. Meanwhile, all five ERF genes in larch are positioned on distant branches within their respective clusters, a result of the independent evolution of gymnosperms and angiosperms over a span of 300 million years. In comparison, the high similarity between *ERF3-like1* in larch and *ERF1* in *Pinus tabuliformis* indicates the relatively conserved evolution of ERF genes within gymnosperms.

The initiation of flowering in angiosperms is primarily regulated by photoperiodic pathway, vernalization pathway, age pathway, temperature pathway, and gibberellin pathway. These flowering pathways also exist in gymnosperms [12]. Numerous studies have shown that ERF genes can respond to environmental signals to regulate plant flowering. For instance, the ERF transcription factor *TOE4b* in soybean (*Glycine max*) can respond to photoperiod and delay flowering [20]. The ERF transcription factor *LATE FLOWERING SEMI-DWARF* (*LSF*) in rice (*Oryza sativa*) responds to long photoperiods and induces flowering by removing the suppressive effect [21]. *LlERF110* in lily is rapidly induced by high temperature, and its overexpression reduces plant heat tolerance while simultaneously suppressing key flowering genes *AtFUL*, *AtAP1*, *AtLFY*, and *AtSOC1*, resulting in delayed bolting in *Arabidopsis* [22, 29]. *CmERF110* in *Chrysanthemum morifolium*responds to photoperiodic signals and interacts with the key factor *FLK* in the autonomous pathway to regulate chrysanthemum flowering. Heterologous expression studies have demonstrated its ability to restore the phenotype of Arabidopsis mutants, suggesting the existence of similar flowering regulatory mechanisms in plants [25]. In this study, these five genes exhibit remarkable consistency in their response to temperature, with high expression levels observed under low-temperature conditions. Interestingly, these genes are also highly expressed in response to ABA, a hormone that primarily regulates dormancy [33]. Accordingly, we speculate that these genes in the shoot apex can respond to accumulated temperature signals, breaking dormancy and initiating reproductive growth. Regarding the response to photoperiod, *ERF3-like1* shows high expression under short-day conditions, while *ABR1-like* and *ERF071* exhibit low expression. The other two genes do not appear to be sensitive to changes in photoperiod. In the local larch population, flowering induction mainly occurs one month after the summer solstice when the daylight duration gradually decreases. This may suggest that *ABR1-like* and *ERF071* can perceive short-day signals and relieve the inhibition of flowering. However, the expression pattern of *ERF3-like1* remains difficult to explain. In fact, the differences in gene expression under different photoperiods are much smaller than those observed under different temperature conditions. Therefore, we believe that these genes are more likely to be involved in the temperature pathway rather than the photoperiodic pathway in regulating reproduction in larch.

In the studies related to flowering regulation, ERF genes are mostly involved as flowering repressors [34]. In addition to the genes mentioned earlier, *ERF1* in Arabidopsis acts as a negative regulator of flowering time by directly inhibiting the expression of the flowering locus FT [28]. ERF3 homolog *CmERF3* in chrysanthemum also functions as a floral repressor, cooperating with *CmBBX8* to suppress the expression of *CmFTL1*, thereby regulating the nutritional and reproductive development of chrysanthemums [26]. The involvement of ERF genes in flowering regulation in gymnosperms is poorly documented, and most of the studies have mainly focused on transcriptomic expression analysis due to the lack of reference genomes [35–38]. In our previous research [24], RNA-seq data revealed a consistent trend among these five genes: they exhibited lower expression levels in buds of the seed high-yield clone (Dh_B, Fig. 2D), suggesting their inhibitory role in larch seed setting. Furthermore, these genes were not highly expressed in the needles, which are the primary receptors of environmental signals. Therefore, it is highly likely that after the environmental signals are received by the needles, they are transmitted through a series of signal transduction processes to the buds, where they regulate the expression of these genes and flowering. The results of qRT-PCR in this study were consistent with RNA-seq data, with the expression levels in the buds generally higher than those in the leaves, except for *ERF3*. Moreover, the expression levels were higher in male cones compared to female cones, which increases the likelihood of these genes participating in larch reproduction as negative regulators.

Unlike short-lived herbaceous plants, tree flowering exhibits clear age effects [39, 40], manifesting as the vegetative growth of axillary buds in young trees [41]. Only when certain genes are activated (or relieved from inhibition) after reaching a certain age, reproductive growth begins. Studies on the ontogeny transition in gymnosperms have mainly focused on MADS family genes associated with floral organs, such as *DAL1* in pine [11] and larch [39], which have demonstrated significant age effects and can promote early flowering in Arabidopsis. However, there is currently no research available on the flowering determination of mature individuals in gymnosperms. In this study, the genes *ERF3-like1* and *ERF071* in larch exhibited pronounced age effects: almost no expression was observed in immature materials, while significant expression was observed in various tissues of adult plants, with higher expression in reproductive organs compared to vegetative organs. This indicates that these two genes are likely crucial for the development of a sound reproductive regulatory mechanism in adult larch trees.

In some studies, ERF genes appear to be involved in floral sex differentiation. For example, in cucumber(*Cucumis sativus*), the ethylene signaling pathway activates *CsERF31* through *CsEIN3*, which then stimulates *CsACS2*, triggering a positive feedback loop that ensures the development of female flowers instead of hermaphroditic flowers [42]. In our study, these genes showed higher expression levels in male cones compared to female cones. However, it is still unclear whether they are involved in the sex determination of larch cones. Considering the possibility that these genes may function as negative regulators in flowering, it is plausible to speculate that lower expression levels of these genes might be more favorable for larch fruit development. Furthermore, there are some studies suggesting that ERF genes can influence the size of floral organs and fruits [43, 44]. Although this study did not include relevant analyses, it is worth considering this aspect to enhance seed yield.

## Conclusion

In this study, sequence analysis and gene expression pattern analysis were conducted on five ERF genes that may be involved in reproduction regulation in larch. The results showed that these five genes exhibited high homology with *ABR1*, *ERF3*, *ERF071*, and *ERF109* in *Arabidopsis*. Among them, *ERF3-like1* and *ERF071* demonstrated significant differences in expression among samples of different ages, suggesting their involvement in the age pathway of flowering. *LkoABR1* and *LkoERF109* show a significant increase during the flowering induction period. All five genes showed induced upregulation in response to low temperature and ABA, indicating their potential role in regulating larch reproduction through inhibitory effects. Additionally, these genes exhibited lower expression in female cones compared to male cones, suggesting that regulating the expression of these genes may help us improve the reproduction and seed setting of larch.

## Materials and methods

### Source of experimental materials

Embryogenic callus material and 90-day-old seedlings were obtained from our laboratory’s previous preparations and sowing, respectively. The remaining materials were collected from the hybrid larch (*Larix kaempferi × Larix olgensis*) second-generation seed orchard located in Qingshan Forest Farm, Linkou County, Mudanjiang, China (45°24’45.49″ N, 130°32’55.10″ E).

### Bioinformatics analysis

Different plant ERF (Ethylene Response Factor) family sequences were retrieved from the UniProt database (https://www.uniprot.org, 2023-09-15.). Motif analysis was conducted using the MEME online tool (https://meme-suite.org/meme, 2023-10-20.). A phylogenetic tree was constructed using the Neighbor-Joining (NJ) method in MEGA 11.0 software. The physicochemical properties of the sequences and the prediction of secondary/tertiary structures were analyzed using online software (https://prabi.ibcp.fr/htm/site/web/app.php, 2023-10-27.). Interaction networks were predicted and GO enrichment analysis was performed using the String database v12.0 (https://string-db.org, 2023-10-30.), and the interaction networks were visualized using Cytoscape 3.9.1.

### Analysis of expression patterns at different developmental stages and tissues

Embryogenic callus tissue, 90-day-old seedlings, needles of adult plants, female cones, and male cones were collected and ground in liquid nitrogen. Nascent buds and coniferous materials from high-yielding clones and low-yielding clones during the flowering induction period were collected and labeled as CKN (CKN: Needles of low yield clones), DhN (Needs of high yield clones), CKB (Buds of low yield clones) and DhB (Buds of high yield clones) [24]. RNA was extracted for qRT-PCR analysis. Transcriptomic data at different stages of shoot development were obtained from the CNCB database (China National Center for Bioinformation, https://www.cncb.ac.cn/, accession number: CRA007780) [24]. Histograms were plotted using GraphPad Prism 10 software.

### Expression analysis of genes in response to phytohormones

To investigate the gene response to phytohormones, 90-day-old hybrid larch seedlings in normal growth conditions were treated with solutions of 50 mg/L GA_3_, ABA, and IAA, respectively. After 0, 24, 48, and 96 h of treatment, whole seedlings were collected and ground in liquid nitrogen for RNA extraction. The gene expression was analyzed by qRT-PCR. Line graphs were plotted using GraphPad Prism 10 software.

### Expression analysis of genes in response to environmental signals

To investigate the response of genes to environmental signals, 90-day-old hybrid larch seedlings in normal growth were subjected to different light cycles (long-day and short-day) for 15 days to study gene response to photoperiod. Similarly, the seedlings were cultured at room temperature (25 °C) and low temperature (8 °C) for 15 days to study gene response to temperature. Whole seedlings were collected after treatment and RNA was extracted by liquid nitrogen grinding for subsequent qRT-PCR analysis of gene expression. Histograms were generated using GraphPad Prism 10 software.

### Analysis of gene expression by real-time quantitative PCR (qRT-PCR)

Primers for each gene were designed using Primer 6.0 software (see Table 3). RNA was extracted from shoots using the PureLinkTM Plant RNA Reagent Kit (TaKaRa, Dalian, China). Reverse transcription was performed using the PrimeScript RT Kit Perfect Real Time (TaKaRa). The reference gene used was α-tubulin. Fluorescence quantification reagents were obtained from the SYBR Premix Ex TaqII (Tli RNaseH Plus) kit (TaKaRa). Quantitative RT-PCR reactions were carried out using the qTOWER 3G Cycler. The relative expression levels of transcripts were determined using the 2^−ΔΔCT^ method.

**Table 3:** The primers used in real-time fluorescent quantitative PCR.

Gene	Forward and Reverse Primers (5’ -3’)	
*LkoERF109*	TCAAACGATTGCGGGAAGT	CCTGGAATTGGGATGGAGT
*LkoERF3*	CTCCGCCTCCGTTCAAATAC	AGAAGCGCCTTGATCGTTGT
*LkoERF071*	GACGCAAATGAGTCTACCT	GATTAGAACCCACTGACAAC
*LkoERF3-like1*	GTGGATGATGCGGCTACGAT	GAAGGGATTCACGGCAGGAC
*LkoABR1-like*	GATGAACGGCACCCTACAA	CCTATCTGGCGGCTCCTAT

## Data Availability

The raw sequence data reported in this paper have been deposited in the Genome Sequence Archive (Genomics, Proteomics & Bioinformatics 2021) in National Genomics Data Center (Nucleic Acids Res 2022), China National Center for Bioinformation / Beijing Institute of Genomics, Chinese Academy of Sciences (GSA: CRA007780) that are publicly accessible at https://ngdc.cncb.ac.cn/gsa.
